# Replacement of polyps with type 1 macular neovascularization in polypoidal choroidal vasculopathy imaged with swept source OCT angiography

**DOI:** 10.1016/j.ajoc.2021.101057

**Published:** 2021-03-11

**Authors:** Mengxi Shen, Qiyu Bo, Minlu Song, Xiaoshuang Jiang, Zohar Yehoshua, Giovanni Gregori, Xiaodong Sun, Fenghua Wang, Philip J. Rosenfeld

**Affiliations:** a Department of Ophthalmology, Bascom Palmer Eye Institute, University of Miami Miller School of Medicine, Miami, FL, USA; b Department of Ophthalmology, Shanghai General Hospital, Shanghai Jiao Tong University School of Medicine, Shanghai, China; c Department of Ophthalmology, West China Hospital, Sichuan University, Chengdu, China; d National Clinical Research Center for Eye Diseases, China; e Shanghai Key Laboratory of Ocular Fundus Diseases, China; f Shanghai Engineering Center for Visual Science and Photomedicine, China; g Shanghai Engineering Center for Precise Diagnosis and Treatment of Eye Diseases, China

**Keywords:** Anti-vascular endothelial growth factor treatment, Polypoidal choroidal vasculopathy, Polyp, Swept source OCT angiography, Type 1 macular neovascularization

## Abstract

**Purpose:**

To investigate the morphological changes of polyps in eyes with polypoidal choroidal vasculopathy (PCV) after treatment with vascular endothelial growth factor (VEGF) inhibitors using swept source optical coherence tomography angiography (SS-OCTA).

**Observations:**

Following anti-VEGF therapy, polyps were found to evolve into typical type 1 macular neovascularization (MNV) in five eyes. In all of these five eyes, a polypoidal lesion was detected adjacent to a serous or hemorrhagic retinal pigment epithelial detachment (PED).

**Conclusions and importance:**

Polypoidal lesions in PCV can evolve into typical type 1 MNV. This morphological evolution suggests that these polyps are clusters of tangled vessels that can proliferate into a more typical neovascular pattern, and this evolution may be facilitated by being adjacent to a PED. Since this morphological appearance could be associated with a better prognosis, SS-OCTA might be helpful in identifying cases of transformed polyps that may be associated with a decreased risk for vision loss.

## 1 Introduction

Polypoidal choroidal vasculopathy (PCV) is a subtype of neovascular age-related macular degeneration that affects a younger population. PCV is defined by the presence of type 1 macular neovascularization (MNV) characterized by branching vascular networks (BVNs) with polyp-like extensions.^1^ The natural course and prognosis of PCV is highly variable.^2^^,^^3^ Some eyes with symptomatic PCV have a relatively benign course without treatment, and some even experience vision improvement.^4^ While in other cases, the disease progresses, and these polypoidal lesions can cause massive macular hemorrhages resulting in severe vision loss.^5^ The regression of polypoidal lesions is a promising sign in cases with better visual acuity outcomes and may serve as a positive prognostic indicator, while persistent clusters of grape-like polyps have a higher risk of bleeding, leakage, and worse visual outcomes.^6^^,^^7^

Until the advent of optical coherence tomography angiography (OCTA), indocyanine green angiography (ICGA) was the gold standard for diagnosing PCV since Yannuzzi et al. first described the condition in 1990.^8^^,^^9^ Currently, OCTA has revolutionized the way we follow up and manage patients with PCV because it is non-invasive, fast, safe, and easily repeatable.^10^ Compared with spectral domain OCTA (SD-OCTA), the longer wavelength used in swept source OCTA (SS-OCTA) results in improved lesion detection and allows for the detailed visualization of the polypoidal lesions and the BVNs.^11^^,^^12^ The presence of BVN is thought to be synonymous with typical type 1 MNV.

Controversy persists as to whether the internal structure of polypoidal lesions represents aneurysmal diliatations, a tangled vascular network, or even a mixture of both morphologies.^13^, ^14^, ^15^ However, when following eyes with PCV after anti-vascular endothelial growth factor (VEGF) treatment, we observed an evolution in the appearance of some polypoidal lesions in response to treatment. In this case series, we used SS-OCTA to detect a morphological evolution of polypoidal lesions into a more typical appearance for type 1 MNV, and this evolution may be associated with improved prognosis in PCV eyes after anti-VEGF treatment.

## 2 Methods

SS-OCTA imaging (PLEX Elite 9000, Carl Zeiss Meditec, Dublin, CA) was performed using an instrument with a central wavelength of 1060 nm and a scanning rate of 100,000 A-scans per second. The choice of scanning pattern was at the discretion of the treating retina specialist and both 6 × 6 mm and 12 × 12 mm scan patterns were chosen to optimize the visualization of the entire PCV lesion. Both of these scan patterns consist of 500 A-scans per B-scan at 500 B-scan positions and each B-scan was repeated twice at each position, resulting in an A-scans and B-scans separation of 12 μm in 6 × 6 mm images and 24 μm in 12 × 12 mm images. FastTrac motion correction software was used while the images were acquired.

The RPE to RPE-fit slab was used to view the PCV complex. The built-in SS-OCTA segmentation editing software was used to manually correct the segmentation boundaries if needed. When using the RPE to RPE-fit slab, we reviewed the en face angiographic and structural images, as well as the corresponding B-cans, to detect both the flow and structural profiles consistent with a PCV lesion. Polyps were recognized as flow signals underlying a dome-shaped peaked retinal pigment epithelial detachment (PED) on B-scans with en face images corresponding to a configuration previously observed using en face ICGA imaging. BVNs were recognized as vascular patterns on en face flow and structural images corresponding to the double-layer sign with a flow signal detected on the cross-sectional B-scans.

The criteria for reinjection was based on the persistence, increase, or recurrence of intraretinal and/or subretinal fluid on structural OCT. Eyes were considered to have a good prognosis based on the absence or decrease of macular fluid on structural OCT scans and based on their final vision compared with the baseline vision.

## 3 Findings

### 3.1 Case 1

**Fig. 1:**
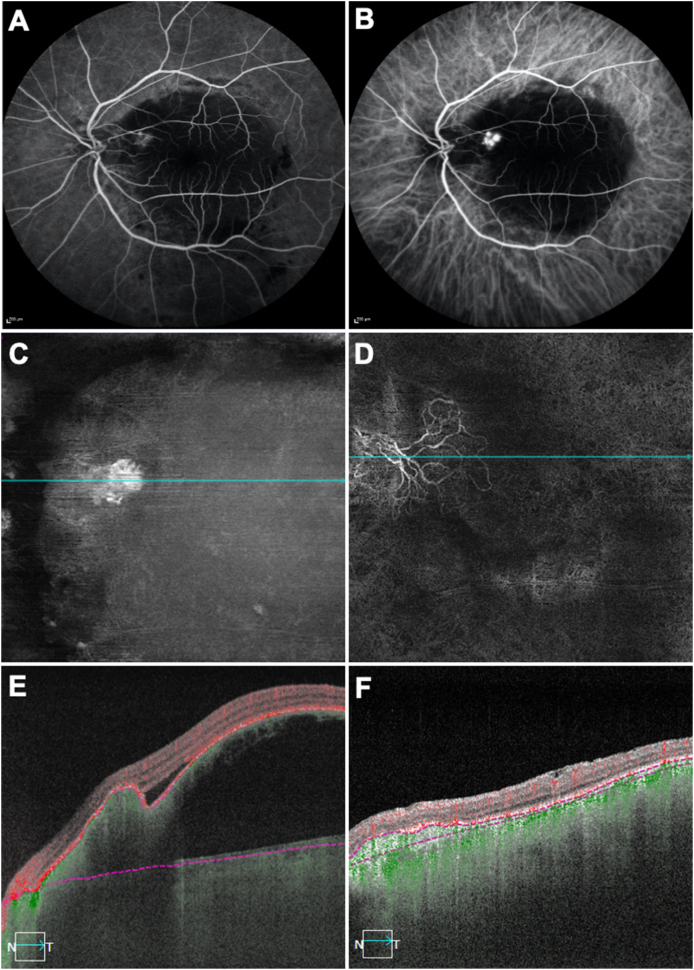
**Multimodal imaging of the left eye of a 57-year-old Hispanic man with polypoidal choroidal vasculopathy over 3 years.** The patient's left eye received 15 injections previously, BCVA 20/400. A) Fluorescein angiography (57 seconds) showing a hyperfluorescence in a hemorrhagic retinal pigment epithelial detachment (PED). B) Indocyanine green angiography (57 seconds) showing a cluster of multi-focal hyperfluorescence, consistent with polyps. C, E) After three more injections, swept source optical coherence tomography angiography (SS-OCTA) *en face* flow image and corresponding cross-sectional B-scan image showing the polypoidal lesion as a dense round structure underneath the PED with a notch, BCVA 20/150. D, F) After 26 more injections over the following three years, SS-OCTA *en face* flow image and corresponding cross-sectional B-scan image showing that the polypoidal lesion grew into a loose and flat vessel structure under the shallow irregular PED, BCVA 20/70. (For interpretation of the references to color in this figure legend, the reader is referred to the Web version of this article.)

A 57-year-old Hispanic man with a diagnosis of PCV in his left eye presented with a new onset hemorrhage. The visual acuity (VA) of the left eye was 20/400. His left eye had received 15 previous anti-VEGF injections before both fluorescein angiography (FA) and ICGA imaging were performed (Fig. 1 A, B). FA confirmed a hemorrhagic PED adjacent to an area of hyperfluorescent leakage and ICGA imaging showed that this area of hyperfluorescence corresponded to a cluster of multi-focal hyperfluorescent polyps consistent with PCV (Fig. 1B). After three monthly injections of anti-VEGF therapy (aflibercept), vision improved to 20/150. At this time the SS-OCTA *en face* flow image and the corresponding cross-sectional B-scan showed the polypoidal lesion as a dense round vascular structure underneath the margin of the retinal pigment epithelial detachment (PED) (Fig. 1 C, E). The left eye received 26 additional aflibercept injections over the following three years and the vision improved to 20/70. The most recent SS-OCTA image showed that the polypoidal lesion evolved into a shallow irregular PED, with the typical appearance of a double-layer sign corresponding to a BNV or type 1 MNV (Fig. 1 D, F).

### 3.2 Case 2

**Fig. 2:**
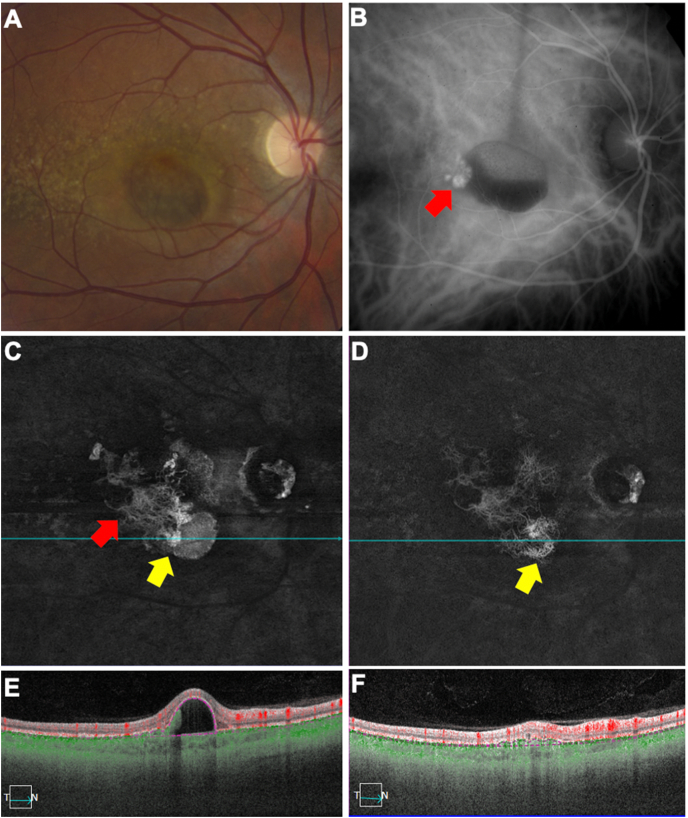
**Multimodal imaging of the right eye of a 38-year-old Caucasian woman with polypoidal choroidal vasculopathy over 12 years.** A) Color fundus imaging of the treatment-naïve right eye in 2008, VA 20/30. B) Indocyanine green angiography (1 minutes and 22 seconds) showing a polypoidal lesion (red arrow) in 2008. C, E) After 28 injections since 2008, swept source optical coherence tomography angiography (SS-OCTA) *en face* flow image showing the polypoidal lesion transformed into a delicate vascular structure (red arrow) in the region previously occupied by the pigment epithelial detachment (PED) within 8 years. Meanwhile, the corresponding cross-sectional B-scan image showing a new polypoidal lesion with a PED at the margin of the neovascular complex (yellow arrow), VA 20/40. D, F) After 26 more injections since 2016, SS-OCTA *en face* flow image and corresponding cross-sectional B-scan image showing that the new polypoidal lesion grew into a low-lying type 1 neovascular network (yellow arrow) within 3.5 years, VA 20/25. (For interpretation of the references to color in this figure legend, the reader is referred to the Web version of this article.)

A 38-year-old Caucasian woman was diagnosed as PCV in her right eye in 2008 with a visual acuity of 20/30. Her right eye was treatment-naïve at presentation. The color fundus imaging in 2008 showed an orange nodule and an adjacent hemorrhagic PED (Fig. 2 A). ICGA at that time confirmed the existence of the polypoidal lesion (Fig. 2 B). In June 2016, after receiving 28 bevacizumab injections, SS-OCTA images showed that the polypoidal lesion had extended beneath the PED and had grown as a delicate vascular network over the previous 8 years (Fig. 2 C, E). In addition, a new polypoidal lesion with an adjacent PED was detected at the margin of the neovascular complex. The vision in right eye was 20/40. In February 2020, after 26 more injections (3 bevacizumab and 23 aflibercept), the vision improved to 20/25. SS-OCTA images showed a low-lying type 1 neovascular network at the location where the polypoidal lesion had existed four years before (Fig. 2 D, F).

### 3.3 Case 3

**Fig. 3:**
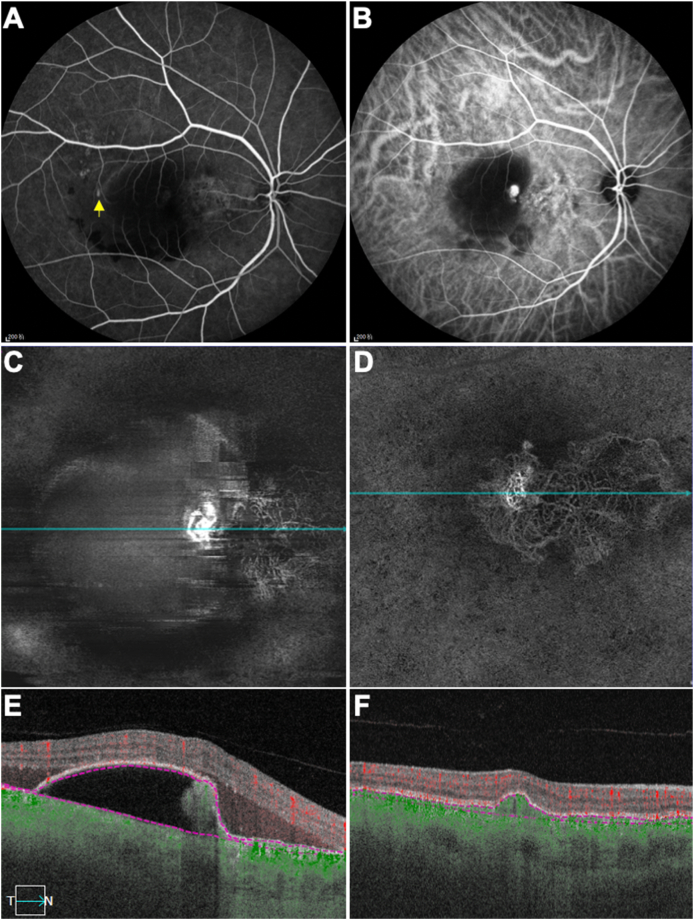
**Multimodal imaging of the right eye of a 67-year-old Asian man with polypoidal choroidal vasculopathy over 13.5 months.** The patient was diagnosed as central serous retinopathy 18 years ago with spontaneous relief. A) Fluorescein angiography (28 seconds) of the treatment-naïve right eye. The yellow arrow indicates where the smokestack plume starts. B) Indocyanine green angiography (3 minutes and 8 seconds) of the treatment-naïve right eye showing a focal hyperfluorescence indicating the polypoidal lesion. C, E) Swept source optical coherence tomography angiography (SS-OCTA) *en face* flow image and corresponding cross-sectional B-scan image of the treatment-naïve right eye showing the polypoidal lesion as a tangled vessel structure underneath the pigment epithelial detachment (PED), BCVA 20/30. D, F) After 3 injections, SS-OCTA *en face* flow image and corresponding cross-sectional B-scan image showing that the polypoidal lesion extended underneath PED and grew into a complicate and flat neovascular lesion within 13.5 months, BCVA 20/20. (For interpretation of the references to color in this figure legend, the reader is referred to the Web version of this article.)

A 67-year-old Asian man complained of blurry vision in his treatment-naïve right eye, which had been diagnosed 18 years previously with central serous chorioretinopathy (CSCR) with spontaneous resolution. His vision was now 20/30 in the right eye. FA and ICGA were performed to establish the diagnosis (Fig. 3 A, B). The early phase FA showed a subtle smokestack plume consistent with CSCR. However, ICGA showed focal hyperfluorescence consistent with a polypoidal lesion at the nasal margin of the PED. The SS-OCTA *en face* flow image and the corresponding cross-sectional B-scan image showed the polypoidal lesion as a tangled vascular structure beneath the PED (Fig. 3 C, E). The patient was given three anti-VEGF injections (ranibizumab) on a monthly basis and then observed for 11.5 months. His vision improved to 20/20 in the most recent visit and the SS-OCTA *en face* image showed a vascular network consitent with type 1 MNV (Fig. 3 D, F). The corresponding cross-sectional B-scan showed a typical type 1 neovascular lesion under a low-lying PED.

### 3.4 Case 4

**Fig. 4:**
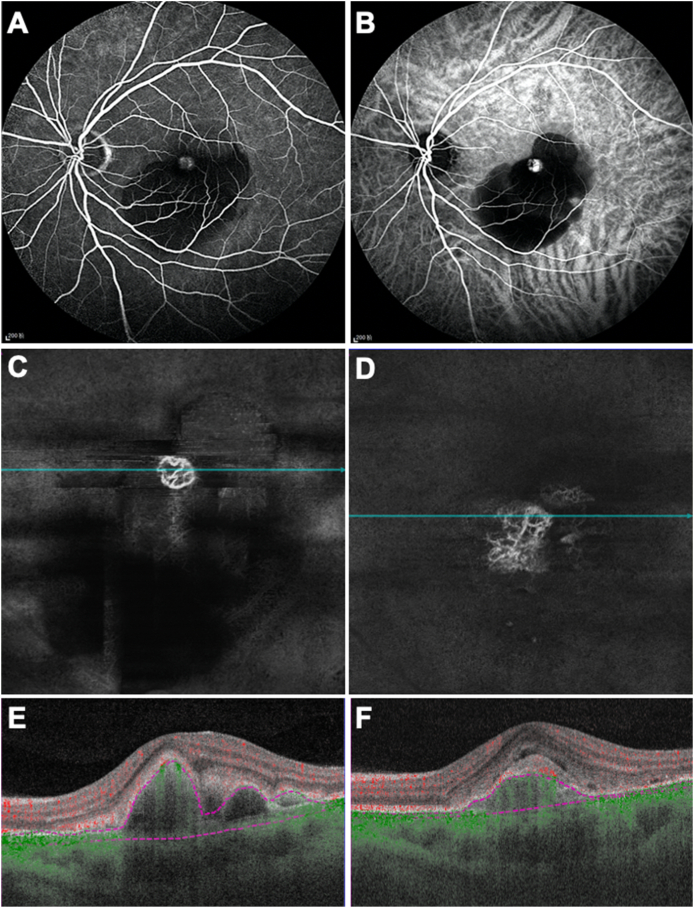
**Multimodal imaging of the left eye of a 58-year-old Asian man with polypoidal choroidal vasculopathy over 7 months.** A) Fluorescein angiography (1 minutes and 29 seconds) of the treatment-naïve left eye. B) Indocyanine green angiography (1 minutes and 29 seconds) of the treatment-naïve left eye showing a focal hyperfluorescence indicating the polypoidal lesion. C, E) Swept source optical coherence tomography angiography (SS-OCTA) *en face* flow image and corresponding cross-sectional B-scan image of the treatment-naïve left eye showing the polypoidal lesion as a round tangled vessel structure in a sharp pigment epithelial detachment (PED), BCVA 20/100. D, F) After 3 injections, SS-OCTA *en face* image and corresponding cross-sectional B-scan image showing that the sharp polypoidal lesion evolved into a flat typical type 1 neovascular lesion within 7 months, BCVA stable at 20/100. (For interpretation of the references to color in this figure legend, the reader is referred to the Web version of this article.)

A 58-year-old Asian man presented with the complaint of vision loss and distortion in his left eye. The VA in the left eye was 20/100. His left eye was treatment naive. ICGA confirmed the diagnosis of PCV, showing focal hyperfluorescence consistent with a polypoidal lesion (Fig. 4 B). The SS-OCTA *en face* flow image showed the polypoidal lesion as a round tangled vascular structure and the corresponding cross-sectional B-scan image showed the sharply peaked PED consistent with a polyp (Fig. 4 C, E). This patient was given three conbercept injections on a monthly basis then observed for five months. The most recent SS-OCTA images (Fig. 4 D, F) showed that the polypoidal lesion had evolved into a more typical type 1 fibrovascular PED with the stable vision of 20/100.

### 3.5 Case 5

**Fig. 5:**
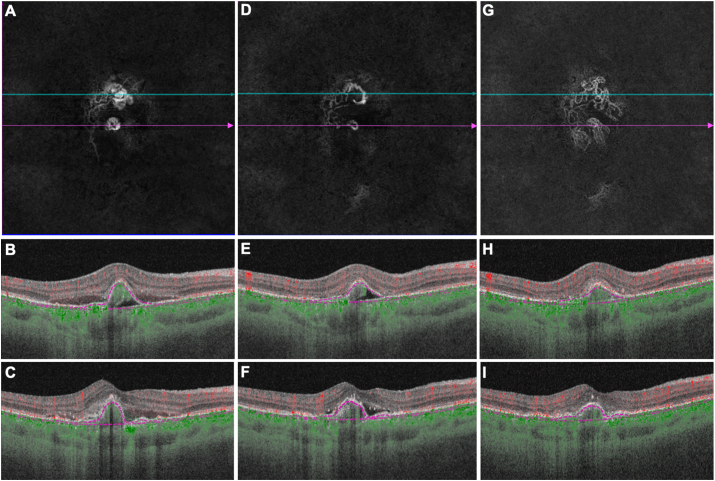
**Swept source optical coherence tomography angiography (SS-OCTA) imaging of the right eye of a 67-year-old Asian man with polypoidal choroidal vasculopathy over 10 months.** A-C) SS-OCTA *en face* flow image and corresponding cross-sectional B-scan images (B corresponds to blue line in A; C corresponds to pink line in A) of the treatment-naïve right eye showing two polypoidal lesions as a round tangled vessel structure in a sharp pigment epithelial detachment (PED), BCVA 20/200. D-F) After 3 injections, SS-OCTA *en face* flow image and corresponding cross-sectional B-scan images (E corresponds to blue line in D; F corresponds to pink line in D) showing the regression of the two polypoidal lesions, BCVA 20/100. G-I) After one more injection, SS-OCTA *en face* flow image and corresponding cross-sectional B-scan images (H corresponds to blue line in G; I corresponds to pink line in G) showing that the two sharp polypoidal lesions grew into a more typical type 1 neovascular lesion without subretinal fluid within 10 months, BCVA 20/30. (For interpretation of the references to color in this figure legend, the reader is referred to the Web version of this article.)

A 67-year-old Asian man complained of acute loss of vision in his right eye. ICGA was not performed since the patient was allergic to iodine. He had not received any prior treatment to his right eye. SSOCTA imaging confirmed the diagnosis of PCV, with two polypoidal configurations detected on the *en face* flow image corresponding to sharply peaked PEDs on the cross-sectional B-scans (Fig. 5 A, B, C). In addition to the polyps, a double-layer sign with flow signal could be detected on the cross-sectional B scans consistent with type 1 MNV or a BVN, which also supported the diagnosis of PCV. His vision was 20/200 at presentation. After three consecutive monthly anti-VEGF injections (conbercept), his vision improved to 20/100. The SS-OCTA images showed a morphological change in the two polypoidal lesions (Fig. 5 D, E, F). Since there was still some subretinal fluid, another conbercept injection was given and then observed for another 6.5 months. At this time, the vision in his right eye had improved to 20/30. The SS-OCTA images showed a more typical type 1 neovascular lesion without subretinal fluid (Fig. 5 G, H, I).

## 4 Discussion

**Fig. 6:**
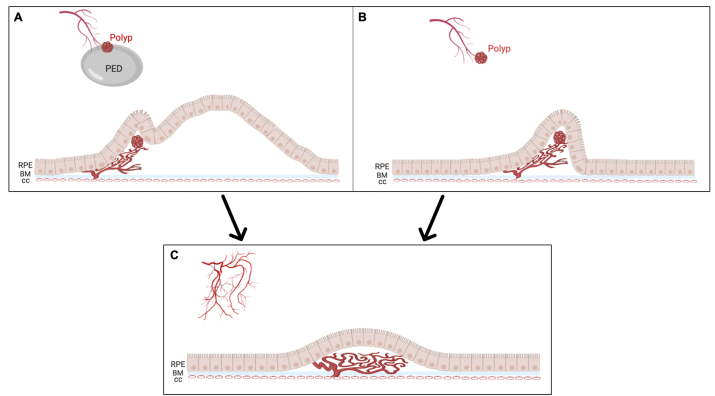
**A schematic showing the evolution of a polypoidal lesion in polypoidal choroidal vasculopathy (PCV) cases into type 1 macular neovascularization (MNV).** A) The polypoidal lesion is located adjacent to a serous or hemorrhagic retinal pigment epithelial detachment (PED). B) As the fluid resolves after anti-vascular endothelial growth factor (VEGF) treatment, the polypoidal lesion is more easily seen to sprout from a branching vascular network (BVN) and is contained within a sharply peaked PED. C) After additional anti-VEGF therapy, the polypoidal lesion evolves into typical type 1 MNV. RPE - retinal pigment epithelium, BM - Bruch's membrane, cc - choriocapillaris. Created with BioRender.com.

In this case series, we described five eyes in which the polypoidal lesion evolved into a more typical appearance for type 1 MNV after anti-VEGF treatment as shown by our diagrammatic representation in Fig. 6. In all five eyes, the vision was stable or even improved. The natural course of PCV has been reported to follow a remitting-relapsing course with long-term preservation of good vision in nearly two-thirds of patients,^16^ while the remaining one third of the patients may suffer from massive submacular hemorrhages within 10 years, even with the proper treatment after initial presentation.^5^ In a natural history study of PCV eyes that were never treated, Uyama et al. reported that the polypoidal lesion resolved spontaneously in three out of fourteen eyes (21%) within an average of 39.9 months follow up.^6^ However, they described that these lesions disappeared completely on ICGA, which likely corresponded to the evolution of a polypoidal lesion to a low-lying PED or type 1 MNV as we describe in our current study. This may have been difficult to appreciate on early stage ICGA when most polyps are visualized.

The regression of polyps on ICGA is a crucial endpoint in most of the clinical trials related to PCV.^17^^,^^18^ The rate of complete polypoidal lesion regression at month 24 was reported to be 26.7% after ranibizumab monotherapy in Everest II study.^19^ In a study reported by Kang et al.,^3^ ICGA images in their Fig. 5 showed a case of in which the original polyp regressed followed by new polyps arising during 37 months follow up. They also found that 22.2% of the eyes had polyp regression based on ICGA imaging three months after anti-VEGF injections. Morever, Lee et al. reported 66.7% of the treatment-naïve PCV eyes had complete polyp regression based on ICGA imaging one year after bi-monthly aflibercept.^20^ In their study, a series of ICGA images (their Fig. 6) demonstrated the regression of the polyps. However, it is hard to see what neovascular structure remained when the polyps regressed on ICGA imaging. Our findings would be consistent with polyp regression on ICGA imaging; however, we propose that type 1 MNV or BVN replaces these polyps. This evolution of a polypoidal lesion may provide valuable information regarding disease activity in PCV eyes and may be a positive prognostic sign. Future clinical trials should consider including SS-OCTA findings as one of the endpoints.

In our current series of five eyes, the polypoidal lesion evolved into a typical type 1 MNV after multiple anti-VEGF injections, which supports the idea that the polypoidal lesion is a tangled vascular structure, instead of an aneurysmal lesion. It is unlikely that an aneurysmal lesion would respond to anti-VEGF treatment by evolving into type 1 MNV, but a tangled neovacular structure may respond by remodeling under the influence of anti-VEGF injections, similar to the vascular remodeling of typical type 1 MNV.^21^ Whether polyps with a tangled vascular structure are associated with a better prognosis requires further investigation. Why all eyes with polyps don't have similar changes and whether these other eyes had polyps with more of an aneurysmal appearance remains to be determined.

## 5 Conclusions

SS-OCTA imaging was able to clearly visualize the detailed structure of PCV without the use of ICGA and characterize the evolution of polyps into typical type 1 MNV after anti-VEGF therapy. All five eyes had stable or even improved vision outcomes. Since this morphological change may be associated with improved prognosis in vision outcomes, SS-OCTA should be helpful in identifying those PCV cases in which the eyes are at greater risk for vision loss after anti-VEGF therapy. Further SS-OCTA studies are needed to investigate the evolution, natural history, and response of PCV to anti-VEGF therapy.

### Patient consent

Written informed consent was obtained from patients for publication of these case reports and any accompanying images.

## Financial support

Research supported by grants from Carl Zeiss Meditec, Inc. (Dublin, CA), the Salah Foundation, an unrestricted grant from the Research to Prevent Blindness, Inc., New York, NY, and the National Eye Institute Center Core Grant (P30EY014801) to the Department of Ophthalmology, 10.13039/100006686University of Miami Miller School of Medicine. The funding organizations had no role in the design or conduct of this research.

## Financial disclosure(s)

The author(s) have made the following disclosures**:**

Xiaodong Sun is a consultant for Novartis, Bayer, Chengdu Kanghong Biotech, Roche and Alcon.

Giovanni Gregori receives research support from Carl Zeiss Meditec, Inc, and co-owns a patent with the University of Miami that is licensed to Carl Zeiss Meditec, Inc.

Philip Rosenfeld receives research support from Carl Zeiss Meditec, Inc and Stealth Bio Therapeutics. He is a consultant for Apellis, Biogen, Boehringer-Ingelheim, Carl Zeiss Meditec, Chengdu Kanghong Biotech, EyePoint, Ocunexus Therapeutics, Ocudyne, and Unity Biotechnology. He has equity interests in Apellis, Ocudyne, Valitor, and Verana Health.

The following authors have no financial disclosures: MS, QB, MS, XJ, ZY, FW.

## Authorship

All authors attest that they meet the current ICMJE criteria for Authorship.

## Institutional review board approval statement

This study was approved by the Institutional Review Boards at both the Shanghai General Hospital and the University of Miami Miller school of Medicine.
